# Supplementary material for: Small-Angle X-ray Scattering Models of APOBEC3B Catalytic Domain in a Complex with a Single-Stranded DNA Inhibitor
Source: Viruses. 2021 Feb 12;13(2):290. doi: 10.3390/v13020290 (PMC7918907; doi:10.3390/v13020290)
Supplement: Supplementary file 1 [file viruses-13-00290-s001.pdf]

Supplementary Material

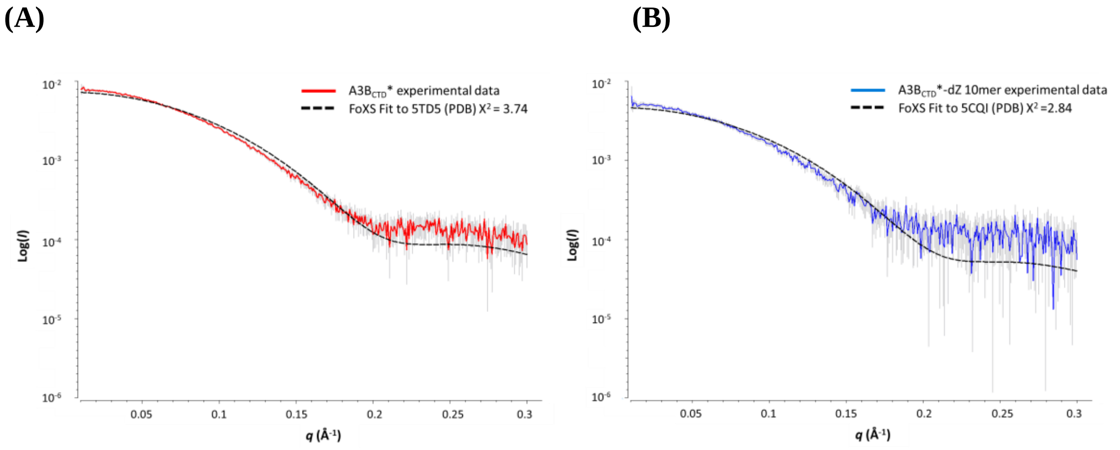

Figure S1. Fitting of A3BCTD* and A3BCTD*/dZ-oligo SAXS profiles to A3BCTD-ssDNA (5TD5*) and A3BCTD (5CQI) crystal structures, respectively**.** Model fit of ssDNA-bound 5TD5* to ligand-free A3BCTD* scattering data (left), fit of ligand-free 5CQI to A3BCTD*/dZ-oligo (1 to 2 ratio) scattering data.

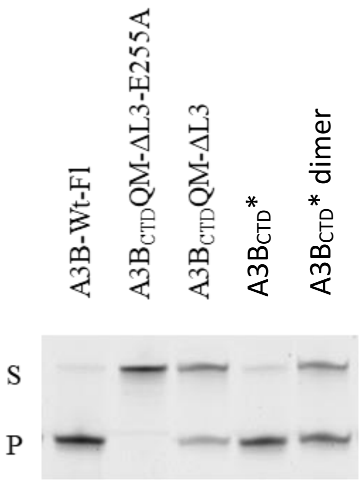

Figure S2. *In vitro* deaminase activity assay of the A3BCTD variants. An established *in vitro* DNA deamination in-gel based assay (2) was performed using a final concentration of 5 µM of our purified A3B variants along with 800 nM of a fluorescein-tagged oligonucleotide (TC 3' 6-FAM). The wild type full-length A3B (A3B-Wt-Fl) protein was used as a control of catalytic activity. S denotes supernatant fraction, P denotes pellet fraction.

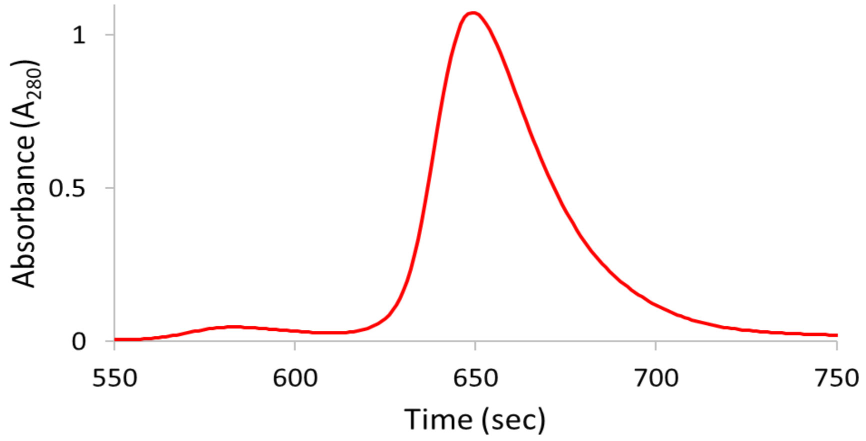

Figure S3. Size exclusion chromatography elution profile of A3B$_{CTD}$*.

Analysis of A3B$_{CTD}$* (10 mg/mL) using SEC-FPLC in pH 5.5 buffer. Sample was run at 25 °C through a SEC column at a flow rate of 0.2 mL/min and monitored at 280 nm (A_{280}).

Table S1. Interface residue contacts of A3B$_{CTD}$-QM-ΔL3 (5CQI, PDB) interface A (Δ*G* = -12.65 kcal/mol) (PRISM (74))

Number	A3B$_{CTD}$_A	Residue contact	A3B$_{CTD}$ _B
1	pdb1_A_TYR_350	←→	pdb2_B_ASP_194
2	pdb1_A_TYR_215	←→	pdb2_B_TYR_191
3	pdb1_A_SER_264	←→	pdb2_B_SER_264
4	pdb1_A_SER_264	←→	pdb2_B_VAL_262
5	pdb1_A_PRO_263	←→	pdb2_B_PRO_263
6	pdb1_A_SER_264	←→	pdb2_B_ASP_260
7	pdb1_A_SER_264	←→	pdb2_B_LEU_261
8	pdb1_A_GLN_233	←→	pdb2_B_GLU_241
9	pdb1_A_ASP_232	←→	pdb2_B_GLU_241
10	pdb1_A_LEU_261	←→	pdb2_B_SER_264
11	pdb1_A_TYR_191	←→	pdb2_B_PHE_348
12	pdb1_A_ASP_260	←→	pdb2_B_GLN_266
13	pdb1_A_GLN_266	←→	pdb2_B_ASP_260
14	pdb1_A_MET_193	←→	pdb2_B_TYR_191
15	pdb1_A_SER_264	←→	pdb2_B_PRO_263
16	pdb1_A_ASP_194	←→	pdb2_B_TYR_350
17	pdb1_A_GLN_233	←→	pdb2_B_ARG_257
18	pdb1_A_LEU_192	←→	pdb2_B_TYR_191

19	pdb1_A_TYR_191	←→	pdb2_B_TYR_191
20	pdb1_A_TYR_191	←→	pdb2_B_MET_193
21	pdb1_A_TYR_191	←→	pdb2_B_LEU_192
22	pdb1_A_TYR_191	←→	pdb2_B_PHE_237

Table S2. Interface residue contacts of A3B$_{CTD}$-QM-ΔL3 (5CQI, PDB) interface B (ΔG = -2.94 kcal/mol) (PRISM [74])

Number	A3B$_{CTD}$_A	Residue contact	A3B$_{CTD}$ _B
1	pdb1_A_GLY_251	←→	pdb2_B_TYR_191
2	pdb1_A_GLN_213	←→	pdb2_B_ASP_194
3	pdb1_A_HIS_253	←→	pdb2_B_TYR_350
4	pdb1_A_ARG_212	←→	pdb2_B_PRO_195
5	pdb1_A_ARG_212	←→	pdb2_B_ASP_194
6	pdb1_A_ARG_212	←→	pdb2_B_THR_197
7	pdb1_A_ARG_212	←→	pdb2_B_ASP_196
8	pdb1_A_SER_250	←→	pdb2_B_MET_193
9	pdb1_A_GLY_251	←→	pdb2_B_LEU_192
10	pdb1_A_GLU_241	←→	pdb2_B_PHE_237
11	pdb1_A_TYR_191	←→	pdb2_B_SER_250
12	pdb1_A_SER_250	←→	pdb2_B_PHE_237
13	pdb1_A_GLY_251	←→	pdb2_B_TYR_350
14	pdb1_A_TRP_287	←→	pdb2_B_TYR_350
15	pdb1_A_TRP_287	←→	pdb2_B_GLN_352

Table S3. Interface residue contacts of A3B$_{CTD}$* interface model 1 (ΔG = -38.13 kcal/mol) (PRISM [74])

Number	A3B$_{CTD}$*_A	Residue contact	A3B$_{CTD}$*_B
1	pdb1_A_ASP_260	←→	pdb2_B_TYR_350
2	pdb1_A_CYS_239	←→	pdb2_B_CYS_239
3	pdb1_A_LYS_213	←→	pdb2_B_LYS_213
4	pdb1_A_TYR_191	←→	pdb2_B_ARG_257
5	pdb1_A_GLU_241	←→	pdb2_B_TYR_215
6	pdb1_A_ASP_196	←→	pdb2_B_SER_250
7	pdb1_A_ARG_257	←→	pdb2_B_THR_197
8	pdb1_A_THR_197	←→	pdb2_B_GLU_241
9	pdb1_A_ASP_260	←→	pdb2_B_ASP_194
10	pdb1_A_ARG_257	←→	pdb2_B_MET_193
11	pdb1_A_TYR_215	←→	pdb2_B_SER_250

Number	A3BCTD*_A	Residue contact	A3BCTD*_B
12	pdb1_A_MET_193	←→	pdb2_B_ARG_257
13	pdb1_A_TYR_191	←→	pdb2_B_LEU_261
14	pdb1_A_PHE_237	←→	pdb2_B_GLU_241
15	pdb1_A_MET_235	←→	pdb2_B_TYR_191
16	pdb1_A_TYR_218	←→	pdb2_B_TYR_191
17	pdb1_A_LEU_261	←→	pdb2_B_TYR_191
18	pdb1_A_LEU_265	←→	pdb2_B_TYR_191
19	pdb1_A_ARG_257	←→	pdb2_B_PHE_237
20	pdb1_A_TYR_215	←→	pdb2_B_GLU_241
21	pdb1_A_MET_193	←→	pdb2_B_GLU_241
22	pdb1_A_THR_197	←→	pdb2_B_SER_250
23	pdb1_A_GLU_241	←→	pdb2_B_THR_197
24	pdb1_A_PHE_237	←→	pdb2_B_ARG_257
25	pdb1_A_ASP_194	←→	pdb2_B_ARG_257
26	pdb1_A_SER_250	←→	pdb2_B_LYS_213
27	pdb1_A_ASP_194	←→	pdb2_B_ARG_252
28	pdb1_A_ARG_257	←→	pdb2_B_ASP_194
29	pdb1_A_SER_264	←→	pdb2_B_ARG_190
30	pdb1_A_SER_264	←→	pdb2_B_TYR_191
31	pdb1_A_SER_264	←→	pdb2_B_LEU_192
32	pdb1_A_TYR_191	←→	pdb2_B_LEU_238
33	pdb1_A_PRO_263	←→	pdb2_B_TYR_350
34	pdb1_A_ASN_201	←→	pdb2_B_SER_250
35	pdb1_A_ARG_252	←→	pdb2_B_ASP_196
36	pdb1_A_HIS_234	←→	pdb2_B_TYR_191

Table S4. Interface residue contacts of A3BCTD* interface model 2 (ΔG = -18.6 kcal/mol) (PRISM [(74]**)**

Number	A3BCTD*_A	Residue contact	A3BCTD*_B
1	pdb1_A_GLY_291	←→	pdb2_B_GLY_288
2	pdb1_A_GLU_299	←→	pdb2_B_ARG_252
3	pdb1_A_GLU_299	←→	pdb2_B_GLY_251
4	pdb1_A_GLU_299	←→	pdb2_B_SER_250
5	pdb1_A_GLY_291	←→	pdb2_B_SER_286
6	pdb1_A_GLY_291	←→	pdb2_B_TRP_287
7	pdb1_A_TRP_287	←→	pdb2_B_ALA_295
8	pdb1_A_ALA_295	←→	pdb2_B_TRP_287

Number	A3B$_{CTD}$*_A	Residue contact	A3B$_{CTD}$*_B
9	pdb1_A_MET_325	←→	pdb2_B_SER_286
10	pdb1_A_ASP_260	←→	pdb2_B_ARG_252
11	pdb1_A_GLU_292	←→	pdb2_B_LEU_256
12	pdb1_A_GLU_292	←→	pdb2_B_ARG_252
13	pdb1_A_PHE_296	←→	pdb2_B_SER_250
14	pdb1_A_PHE_296	←→	pdb2_B_GLY_251
15	pdb1_A_ALA_295	←→	pdb2_B_HIS_253
16	pdb1_A_SER_250	←→	pdb2_B_GLU_299
17	pdb1_A_PRO_263	←→	pdb2_B_SER_250
18	pdb1_A_PHE_285	←→	pdb2_B_PHE_285
19	pdb1_A_GLU_299	←→	pdb2_B_GLU_241
20	pdb1_A_GLU_299	←→	pdb2_B_ASN_240
21	pdb1_A_ASN_300	←→	pdb2_B_SER_250
22	pdb1_A_ASN_300	←→	pdb2_B_GLY_251
23	pdb1_A_GLU_292	←→	pdb2_B_TRP_287
24	pdb1_A_ASP_260	←→	pdb2_B_ASP_260
25	pdb1_A_LEU_256	←→	pdb2_B_GLU_292
26	pdb1_A_LEU_259	←→	pdb2_B_ARG_252
27	pdb1_A_GLY_251	←→	pdb2_B_GLU_299

Table S5. Model 1 dimer interface assessment using PISA [(76] (ΔG = -2.00 kcal/mol, interface area = 1034.4 Å^2)

Number	A3B$_{CTD}$*_A	Residue contact	A3B$_{CTD}$*_B
1	ARG 257	← H-bond →	THR 197
2	THR 197	← H-bond →	GLU 241
3	SER 264	← H-bond →	ARG 190
4	GLU 241	← H-bond →	CYS 239
5	ASP 194	← H-bond →	ARG 252
6	ASP 196	← H-bond →	ARG 252
7	LEU 192	← H-bond →	ARG 257
8	ARG 252	← Salt-bridge →	ASP 196
9	ASP 194	← Salt-bridge →	ARG 252
10	ASP 196	← Salt-bridge →	ARG 252

Table S6. Model 2 dimer interface assessment PISA [(76] (ΔG = -4.00 kcal/mol, interface area = 725.8 Å^2)

Number	A3B$_{CTD}$*_A	Residue contact	A3B$_{CTD}$*_B
1	GLU 292	← H-bond →	ARG 252
2	GLU 299	← H-bond →	SER 250
3	ASP 260	← Salt-bridge →	ARG 252
4	GLU 292	← Salt-bridge →	ARG 252

(A) **(B)**

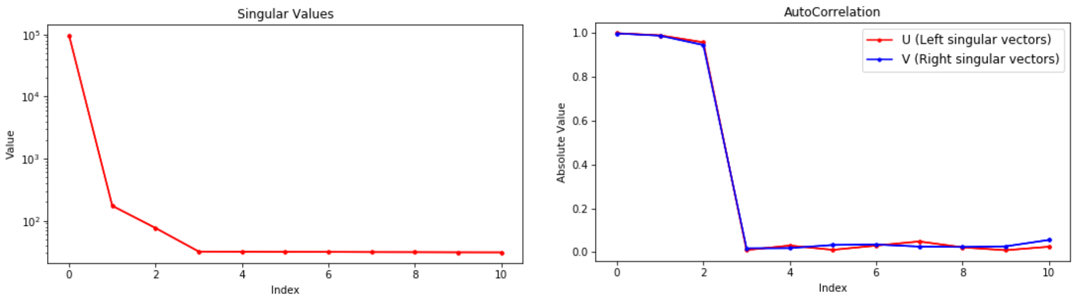

Figure S4. Singular value decomposition (SVD) of A3B$_{CTD}$* dimer with dZ-oligo**.**

SAXS profile assessed using SVD/EFA BioXTAS RAW (1). (A) Represents SVD showing an eigenvalue of three on the index which is equivalent to number of components in the scattering sample. (B) The autocorrelation between the singular vectors (blue and red lines), indicating that the singular values are not so variable.

References

1. Hopkins JB, Gillilan RE, Skou S. BioXTAS RAW: improvements to a free open-source program for small-angle X-ray scattering data reduction and analysis. Journal of Applied Crystallography. 2017;50(5):1545-53.
2. Shi K, Carpenter MA, Kurahashi K, Harris RS, Aihara H. Crystal structure of the DNA deaminase APOBEC3B catalytic domain. J Biol Chem. 2015;290(47):28120-30.
3. Bohn M-F, Shandilya SM, Silvas TV, Nalivaika EA, Kouno T, Kelch BA, et al. The ssDNA mutator APOBEC3A is regulated by cooperative dimerization. Structure. 2015;23(5):903-11.
4. Franke D, Petoukhov M, Konarev P, Panjkovich A, Tuukkanen A, Mertens H, et al. ATSAS 2.8: a comprehensive data analysis suite for small-angle scattering from macromolecular solutions. Journal of Applied Crystallography. 2017;50(4):1212-25.
5. Harris RS, Bishop KN, Sheehy AM, Craig HM, Petersen-Mahrt SK, Watt IN, et al. DNA deamination mediates innate immunity to retroviral infection. Cell. 2003;113(6):803-9.
6. Harris RS. Cancer mutation signatures, DNA damage mechanisms, and potential clinical implications. Genome Medicine. 2013;5(9):1-3.
7. Harris RS, Liddament MT. Retroviral restriction by APOBEC proteins. Nature Reviews Immunology. 2004;4(11):868-77.
8. Jarmuz A, Chester A, Bayliss J, Gisbourne J, Dunham I, Scott J, et al. An anthropoid-specific locus of orphan C to U RNA-editing enzymes on chromosome 22. Genomics. 2002;79(3):285-96.
9. Refsland EW, Harris RS. The APOBEC3 family of retroelement restriction factors. Intrinsic Immunity: Springer; 2013. p. 1-27.
10. Burns MB, Lackey L, Carpenter MA, Rathore A, Land AM, Leonard B, et al. APOBEC3B is an enzymatic source of mutation in breast cancer. Nature. 2013;494(7437):366-70.
11. Burns MB, Temiz NA, Harris RS. Evidence for APOBEC3B mutagenesis in multiple human cancers. Nat Genet. 2013b;45(9):977-83.
12. Law EK, Sieuwerts AM, LaPara K, Leonard B, Starrett GJ, Molan AM, et al. The DNA cytosine deaminase APOBEC3B promotes tamoxifen resistance in ER-positive breast cancer. Science Advances. 2016;2(10):e1601737.
13. Sieuwerts AM, Willis S, Burns MB, Look MP, Meijer-Van Gelder ME, Schlicker A, et al. Elevated APOBEC3B correlates with poor outcomes for estrogen-receptor-positive breast cancers. Hormones and Cancer. 2014;5(6):405-13.
14. Ding Q, Chang C-J, Xie X, Xia W, Yang J-Y, Wang S-C, et al. APOBEC3G promotes liver metastasis in an orthotopic mouse model of colorectal cancer and predicts human hepatic metastasis. J Clin Invest. 2011;121(11).
15. Swanton C, McGranahan N, Starrett GJ, Harris RS. APOBEC enzymes: mutagenic fuel for cancer evolution and heterogeneity. Cancer Discovery. 2015;5(7):704-12.
16. LaRue RS, Jónsson SR, Silverstein KA, Lajoie M, Bertrand D, El-Mabrouk N, et al. The artiodactyl APOBEC3 innate immune repertoire shows evidence for a multi-functional domain organization that existed in the ancestor of placental mammals. BMC Molecular Biology. 2008;9(1):104.
17. LaRue RS, Andrésdóttir V, Blanchard Y, Conticello SG, Derse D, Emerman M, et al. Guidelines for naming nonprimate APOBEC3 genes and proteins. Journal of Virology. 2009;83(2):494-7.
18. Conticello SG. The AID/APOBEC family of nucleic acid mutators. Genome Biology. 2008;9(6):229.
19. Harris RS, Dudley JP. APOBECs and virus restriction. Virology. 2015;479:131-45.
20. Siriwardena SU, Guruge TA, Bhagwat AS. Characterization of the catalytic domain of human APOBEC3B and the critical structural role for a conserved methionine. J Mol Biol. 2015;427(19):3042-55.
21. Fu Y, Ito F, Zhang G, Fernandez B, Yang H, Chen XS. DNA cytosine and methylcytosine deamination by APOBEC3B: enhancing methylcytosine deamination by engineering APOBEC3B. Biochemical Journal. 2015;471(1):25-35.
22. Bonvin M, Greeve J. Effects of point mutations in the cytidine deaminase domains of APOBEC3B on replication and hypermutation of hepatitis B virus in vitro. Journal of General Virology. 2007;88(12):3270-4.
23. Haché G, Liddament MT, Harris RS. The retroviral hypermutation specificity of APOBEC3F and APOBEC3G is governed by the C-terminal DNA cytosine deaminase domain. J Biol Chem. 2005;280(12):10920-4.
24. Hakata Y, Landau NR. Reversed functional organization of mouse and human APOBEC3 cytidine deaminase domains. J Biol Chem. 2006;281(48):36624-31.
25. Navarro F, Bollman B, Chen H, König R, Yu Q, Chiles K, et al. Complementary function of the two catalytic domains of APOBEC3G. Virology. 2005;333(2):374-86.
26. Newman EN, Holmes RK, Craig HM, Klein KC, Lingappa JR, Malim MH, et al. Antiviral function of APOBEC3G can be dissociated from cytidine deaminase activity. Current Biology. 2005;15(2):166-70.

27. Bohn M-F, Shandilya SM, Albin JS, Kouno T, Anderson BD, McDougle RM, et al. Crystal structure of the DNA cytosine deaminase APOBEC3F: the catalytically active and HIV-1 Vif-binding domain. Structure. 2013;21(6):1042-50.
28. Chen K-M, Harjes E, Gross PJ, Fahmy A, Lu Y, Shindo K, et al. Structure of the DNA deaminase domain of the HIV-1 restriction factor APOBEC3G. Nature. 2008;452(7183):116-9.
29. Ko T-P, Lin J-J, Hu C-Y, Hsu Y-H, Wang AH-J, Liaw S-H. Crystal structure of yeast cytosine deaminase insights into enzyme mechanism and evolution. J Biol Chem. 2003;278(21):19111-7.
30. Conticello SG, Thomas CJ, Petersen-Mahrt SK, Neuberger MS. Evolution of the AID/APOBEC family of polynucleotide (deoxy) cytidine deaminases. Molecular Biology and Evolution. 2005;22(2):367-77.
31. Shi K, Carpenter MA, Banerjee S, Shaban NM, Kurahashi K, Salamango DJ, et al. Structural basis for targeted DNA cytosine deamination and mutagenesis by APOBEC3A and APOBEC3B. Nature Structural and Molecular Biology. 2017;24(2):131.
32. Carpenter MA, Rajagurubandara E, Wijesinghe P, Bhagwat AS. Determinants of sequence-specificity within human AID and APOBEC3G. DNA Repair. 2010;9(5):579-87.
33. Kohli RM, Abrams SR, Gajula KS, Maul RW, Gearhart PJ, Stivers JT. A portable hot spot recognition loop transfers sequence preferences from APOBEC family members to activation-induced cytidine deaminase. J Biol Chem. 2009;284(34):22898-904.
34. Kohli RM, Maul RW, Guminski AF, McClure RL, Gajula KS, Saribasak H, et al. Local sequence targeting in the AID/APOBEC family differentially impacts retroviral restriction and antibody diversification. J Biol Chem. 2010;285(52):40956-64.
35. Wang M, Rada C, Neuberger MS. Altering the spectrum of immunoglobulin V gene somatic hypermutation by modifying the active site of AID. The Journal of Experimental Medicine. 2010;207(1):141-53.
36. McDougall WM, Okany C, Smith HC. Deaminase activity on single-stranded DNA (ssDNA) occurs in vitro when APOBEC3G cytidine deaminase forms homotetramers and higher-order complexes. J Biol Chem. 2011;286(35):30655-61.
37. Prohaska KM, Bennett RP, Salter JD, Smith HC. The multifaceted roles of RNA binding in APOBEC cytidine deaminase functions. Wiley Interdisciplinary Reviews: RNA. 2014;5(4):493-508.
38. Salter JD, Bennett RP, Smith HC. The APOBEC protein family: united by structure, divergent in function. Trends in Biochemical Sciences. 2016;41(7):578-94.
39. Smith HC. RNA binding to APOBEC deaminases; Not simply a substrate for C to U editing. RNA Biology. 2017;14(9):1153-65.
40. Zhang W, Zhang X, Tian C, Wang T, Sarkis PTN, Fang Y, et al. Cytidine deaminase APOBEC3B interacts with heterogeneous nuclear ribonucleoprotein K and suppresses hepatitis B virus expression. Cellular Microbiology. 2008;10(1):112-21.
41. Chelico L, Pham P, Calabrese P, Goodman MF. APOBEC3G DNA deaminase acts processively 3′→ 5′ on single-stranded DNA. Nature Structural and Molecular Biology. 2006;13(5):392-9.
42. Holden LG, Prochnow C, Chang YP, Bransteitter R, Chelico L, Sen U, et al. Crystal structure of the anti-viral APOBEC3G catalytic domain and functional implications. Nature. 2008;456(7218):121-4.
43. Li J, Chen Y, Li M, Carpenter MA, McDougle RM, Luengas EM, et al. APOBEC3 multimerization correlates with HIV-1 packaging and restriction activity in living cells. J Mol Biol. 2014;426(6):1296-307.
44. Gallois-Montbrun S, Holmes RK, Swanson CM, Fernández-Ocaña M, Byers HL, Ward MA, et al. Comparison of cellular ribonucleoprotein complexes associated with the APOBEC3F and APOBEC3G antiviral proteins. Journal of Virology. 2008;82(11):5636-42.
45. Cortez LM, Brown AL, Dennis MA, Collins CD, Brown AJ, Mitchell D, et al. APOBEC3A is a prominent cytidine deaminase in breast cancer. PLoS Genetics. 2019;15(12).
46. Lau PP, Zhu H-J, Baldini A, Charnsangavej C, Chan L. Dimeric structure of a human apolipoprotein B mRNA editing protein and cloning and chromosomal localization of its gene. Proceedings of the National Academy of Sciences USA. 1994;91(18):8522-6.
47. Prochnow C, Bransteitter R, Klein MG, Goodman MF, Chen XS. The APOBEC-2 crystal structure and functional implications for the deaminase AID. Nature. 2007;445(7126):447-51.
48. Wedekind JE, Gillilan R, Janda A, Krucinska J, Salter JD, Bennett RP, et al. Nanostructures of APOBEC3G support a hierarchical assembly model of high molecular mass ribonucleoprotein particles from dimeric subunits. J Biol Chem. 2006;281(50):38122-6.
49. Brar SS, Sacho EJ, Tessmer I, Croteau DL, Erie DA, Diaz M. Activation-induced deaminase, AID, is catalytically active as a monomer on single-stranded DNA. DNA Repair. 2008;7(1):77-87.
50. Furukawa A, Nagata T, Matsugami A, Habu Y, Sugiyama R, Hayashi F, et al. Structure, interaction and real-time monitoring of the enzymatic reaction of wild-type APOBEC3G. The EMBO Journal. 2009;28(4):440-51.

51. Kitamura S, Ode H, Nakashima M, Imahashi M, Naganawa Y, Kurosawa T, et al. The APOBEC3C crystal structure and the interface for HIV-1 Vif binding. Nature Structural and Molecular Biology. 2012;19(10):1005-10.
52. Shandilya SM, Nalam MN, Nalivaika EA, Gross PJ, Valesano JC, Shindo K, et al. Crystal structure of the APOBEC3G catalytic domain reveals potential oligomerization interfaces. Structure. 2010;18(1):28-38.
53. Kouno T, Silvas TV, Hilbert BJ, Shandilya SM, Bohn MF, Kelch BA, et al. Crystal structure of APOBEC3A bound to single-stranded DNA reveals structural basis for cytidine deamination and specificity. Nature Communications. 2017;8(1):1-8.
54. Maiti A, Myint W, Kanai T, Delviks-Frankenberry K, Rodriguez CS, Pathak VK, et al. Crystal structure of the catalytic domain of HIV-1 restriction factor APOBEC3G in complex with ssDNA. Nature Communications. 2018;9(1):2460.
55. Harjes S, Jameson GB, Filichev VV, Edwards PJB, Harjes E. NMR-based method of small changes reveals how DNA mutator APOBEC3A interacts with its single-stranded DNA substrate. Nucleic Acids Research. 2017;45(9):5602-13.
56. Byeon I-J.L., Ahn J., Mitra M., Byeon C-H., Herc K., Hritz1 J., et al. NMR structure of human restriction factor APOBEC3A reveals substrate binding and enzyme specificity. Nature Communication 2013;4.
57. Harjes S, Solomon WC, Li M, Chen K-M, Harjes E, Harris RS, et al. Impact of H216 on the DNA binding and catalytic activities of the HIV restriction factor APOBEC3G. Journal of Virology. 2013;87(12):7008-14.
58. Kvach MV, Barzak FM, Harjes S, Schares HA, Jameson GB, Ayoub AM, et al. Inhibiting APOBEC3 activity with single-stranded DNA containing 2'-deoxyzebularine analogs. Biochem. 2019;58(5):391- 400.
59. Mitra M, Hercík K, Byeon I-JL, Ahn J, Hill S, Hinchee-Rodriguez K, et al. Structural determinants of human APOBEC3A enzymatic and nucleic acid binding properties. Nucleic Acids Research. 2014;42(2):1095-110.
60. Barzak FM, Harjes S, Kvach MV, Kurup HM, Jameson GB, Filichev VV, et al. Selective inhibition of APOBEC3 enzymes by single-stranded DNAs containing 2′-deoxyzebularine. Organic and Biomolecular Chemistry. 2019;17(43):9435-41.
61. Ryan TM, Trewhella J, Murphy JM, Keown JR, Casey L, Pearce FG, et al. An optimized SEC-SAXS system enabling high X-ray dose for rapid SAXS assessment with correlated UV measurements for biomolecular structure analysis. Journal of Applied Crystallography. 2018;51(1):97-111.
62. Kirby N, Cowieson N, Hawley AM, Mudie ST, McGillivray DJ, Kusel M, et al. Improved radiation dose efficiency in solution SAXS using a sheath flow sample environment. Acta Crystallographica Section D: Structural Biology. 2016;72(12):1254-66.
63. Mylonas E, Svergun DI. Accuracy of molecular mass determination of proteins in solution by small-angle X-ray scattering. Applied Crystallography. 2007;40(s1):s245-s9.
64. FoXS F. MultiFoXS: single-state and multi-state structural modeling of proteins and their complexes based on SAXS profiles Schneidman-Duhovny, Dina; Hammel, Michal; Tainer, John A.; Sali, Andrej. Nucleic Acids Research. 2016;44:W424-W9.
65. Schneidman-Duhovny D, Hammel M, Tainer JA, Sali A. Accurate SAXS profile computation and its assessment by contrast variation experiments. Biophys J. 2013;105(4):962-74.
66. Betts L, Xiang S, Short SA, Wolfenden R, Carter CW. Cytidine deaminase. The 2·3 Å crystal structure of an enzyme: transition-state analog complex. J Mol Biol. 1994;235(2):635-56.
67. Xiang S, Short SA, Wolfenden R, Carter Jr CW. Transition-state selectivity for a single hydroxyl group during catalysis by cytidine deaminase. Biochem. 1995;34(14):4516-23.
68. Chung SJ, Fromme JC, Verdine GL. Structure of human cytidine deaminase bound to a potent inhibitor. Journal of Medicinal Chemistry. 2005;48(3):658-60.
69. Schrodinger, LLC. The PyMOL Molecular Graphics System, Version 2.1.1. 2015.
70. Bennett RP, Salter JD, Liu X, Wedekind JE, Smith HC. APOBEC3G subunits self-associate via the C-terminal deaminase domain. J Biol Chem. 2008;283(48):33329-36.
71. Kvach MV, Barzak FM, Harjes S, Schares HA, Kurup HM, Jones KF, et al. Differential inhibition of APOBEC3 DNA-mutator isozymes by fluoro- and non-fluoro-substituted 2'-deoxyzebularine embedded in single-stranded DNA. ChemBioChem. 2020;21(7):1028-35.
72. Byeon I-JL, Byeon C-H, Wu T, Mitra M, Singer D, Levin JG, et al. Nuclear magnetic resonance structure of the APOBEC3B catalytic domain: structural basis for substrate binding and DNA deaminase activity. Biochem. 2016;55(21):2944-59.
73. Tuncbag N, Gursoy A, Nussinov R, Keskin O. Predicting protein-protein interactions on a proteome scale by matching evolutionary and structural similarities at interfaces using PRISM. Nature Protocols. 2011;6(9):1341.

74. Baspinar A, Cukuroglu E, Nussinov R, Keskin O, Gursoy A. PRISM: a web server and repository for prediction of protein–protein interactions and modeling their 3D complexes. Nucleic Acids Research. 2014;42(W1):W285-W9.
75. Krissinel E. Crystal contacts as nature's docking solutions. Journal of Computational Chemistry. 2010;31(1):133-43.
76. Krissinel E, Henrick K. Protein interfaces, surfaces and assemblies service PISA at European Bioinformatics Institute. J Mol Biol. 2007;372:774-97.
77. Bechtel TJ, Weerapana E. From structure to redox: The diverse functional roles of disulfides and implications in disease. Proteomics. 2017;17(6):1600391.
78. Chelico L, Sacho EJ, Erie DA, Goodman MF. A model for oligomeric regulation of APOBEC3G cytosine deaminase-dependent restriction of HIV. The Journal of biological chemistry. 2008;283(20):13780-91.
